# Quantification of twenty pharmacologically active dyes in water samples using UPLC-MS/MS

**DOI:** 10.1016/j.heliyon.2022.e09331

**Published:** 2022-04-23

**Authors:** Angelika Tkaczyk-Wlizło, Kamila Mitrowska, Tomasz Błądek

**Affiliations:** Department of Pharmacology and Toxicology, National Veterinary Research Institute (PIWet), Al. Partyzantow 57, Pulawy, Poland

**Keywords:** Malachite green, Methylene blue, Rhodamine, Aquatic environment, Liquid chromatography, Mass spectrometry

## Abstract

This study presents a multi-compound method for the determination of 20 pharmacologically active dyes from 5 different chemical classes in environmental water samples. These compounds, including triphenylmethane dyes (malachite green, crystal violet, brilliant green, ethyl violet, methyl violet 2B, pararosaniline, victoria blue B, victoria blue R, victoria pure blue BO), phenothiazine dyes (methylene blue, azure A, azure B, azure C, new methylene blue, thionine), phenoxazine dye (nile blue A), acridine dyes (acriflavine, proflavine) and xanthene dyes (rhodamine B, rhodamine 6G) constitute pharmacologically active substances (PASs). For the optimisation of sample preparation, different solid-phase extraction (SPE) sorbents and a wide range of pH (from 2 to 12) of water samples were tested. Finally, water samples were preconcentrated and cleaned up on diol SPE cartridges. Extracts were analysed by ultra-performance liquid chromatography-tandem mass spectrometry (UPLC-MS/MS) operating in the positive electrospray ionisation (ESI+) mode. The chromatographic separation of the 20 pharmacologically active dyes was achieved within 5 min by using a pentafluorophenyl (F5) analytical column and mobile phases of ammonium acetate buffer (0.05 M, pH = 3.5) and acetonitrile with gradient elution. The developed method was validated proving to be suitable for the determination of all tested compounds. Limits of quantification were 0.01–0.1 μg/l, are sensitive enough to quantify very low concentration levels of the dyes in environmental water samples. The obtained recovery values for all tested analytes were between 71.2 and 104.9% with a good RSD, less than 14 % at all fortification levels. The application of the developed method to water samples allows the detection of dyes such as crystal violet, rhodamine B, and methyl violet in two wastewater samples in concentration range from 0.017 to 0.0043 μg/l).

## 1 Introduction

There is an increasing interest in the fate of the pharmacologically active substances (PASs) in the aquatic environment. So far, there has been a lot of research published on the occurrence of PASs such as antibiotics, lipid-regulators or nonsteroidal anti-inflammatory drugs but there has been limited data on the presence of the synthetic organic dyes used as PASs in water bodies [1, 2, 3]. Due to colouring properties, synthetic organic dyes are mainly used in textile, tannery, paper and printing industries as well as in the food processing and cosmetic sector. However, some of the synthetic organic dyes are PASs thus, they are used in human and veterinary medicine. In human medicine, they may be used as a component of medicinal products (e.g. acriflavine, methylene blue) or as aseptic agents (e.g. brilliant green, crystal violet, malachite green) [1, 4]. In veterinary medicine, dyes such as malachite green, methylene blue or acriflavine are applied in ornamental fish culture due to their efficiency against pathogenic fungi, protozoan ectoparasites and bacteria [5]. These dyes have never been authorised in the European Union to use in farmed fish intended for human consumption due to their carcinogenetic, mutagenic and teratogenic properties [6]. However, high availability, low cost and most of all high effectiveness against pathogenic fungi, protozoan ectoparasites and bacteria contribute to the fact that these dyes are sometimes illegally used in fish farming [1]. So far, pharmacologically active dyes such as crystal violet, malachite green [7], rhodamine B [8] and rhodamine 6G [8, 9] have been found in the aquatic environmental samples (rivers, groundwater, effluents from waste water treatment plants (WWTPs)) in concentration range from 0.049 to 76.6 μg/l. Additionally, pharmacologically active dyes including brilliant green [10], crystal violet [11], malachite green [12], methylene blue [13, 14] and rhodamine 6G [15] have been found in industrial wastewater (from 55.8 to 1680 μg/l) where the dyes had been used due to its colouring properties. The presence of malachite green, crystal violet [16] as well as brilliant green [17] have been reported also in fish farming water in concentration range from 10 to 83 μg/l.

Up to now, various analytical techniques such as adsorptive stripping voltammetry [18], UV-vis spectrophotometry [19, 20], liquid chromatography with visible and fluorescence detection (LC-vis/FLD) [21], high-performance liquid chromatography with fluorescence detection (HPLC-FLD) [8], liquid chromatography-mass spectrometry (LC-MS) [22] have been applied to determine pharmacologically active dyes in water samples. Due to the low concentration of dyes in water samples, more sensitive apparatuses such as liquid chromatography-tandem mass spectrometry (LC-MS/MS) have been used for their determination [12, 21]. For sample preparation different techniques such as dispersive liquid-liquid microextraction (DLLME) [7], micro-cloud point extraction (MCPE) [23], cloud point extraction (CPE) [19], ultrasound-assisted emulsification liquid-phase microextraction (UA-ELPME) [20], salting-out assisted liquid-liquid extraction (SALLE) [13] and solid-phase extraction (SPE) [8, 21, 24] have been reported. Among sample preparation techniques, SPE has been most often used with the application of sorbents such as Diol for triphenylmethane dyes [21, 25] and Hydrophilic-Lipophilic Balance (HLB) for xanthene dyes [8] determination.

**Figure 1:**
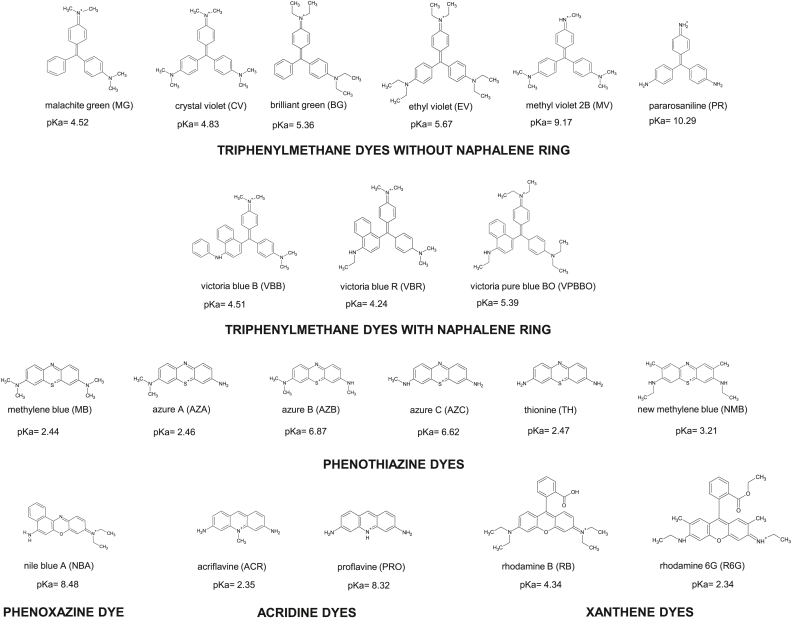
Chemical classes of pharmacologically active dyes.

Most of the aforementioned analytical methods allows the detection of a sole dye in water samples: brilliant green [17], crystal violet [26], malachite green [22], rhodamine B [24], rhodamine 6G [15], two dyes: brilliant green and crystal violet [27], malachite green and crystal violet [7], crystal violet and azure B [28], rhodamine B and rhodamine 6G [8] and up to three dyes: malachite green, crystal violet, rhodamine B [23] and crystal violet, malachite green, methylene blue [29]. Thus, there is a need to develop a new method for the concomitant determining wider, than currently available methods, range of pharmacologically active dyes in water. Our study aimed to develop a simple, fast and reliable method for the determination of 20 pharmacologically active dyes (Figure 1) in different environmental water samples. The merit of the developed method is the possibility to simultaneously analyse 20 pharmacologically active dyes in one analytical approach. Moreover, the application of highly sensitive apparatus such as UPLC-MS/MS allows achieving low limits of detection and quantification of 20 pharmacologically active dyes in water.

## 2 Experimental

### 2.1 Reagents and materials

All chemicals used for LC mobile phases and extraction: glacial acetic acid, acetonitrile and methanol were of LC-MS grade (J. T. Baker, Deventer, the Netherlands). Ultrapure water was of Milli-Q quality (Millipore Corp., Bedford, Ma, USA). L (+) ascorbic acid, ammonium acetate, ammonium formate and, ethanol were obtained from Sigma-Aldrich (Stainheim, Germany). The tested SPE columns were: BAKERBOND spe™ Diol (500 mg, 3 ml; J. T. Baker), CLEAN-UP® Diol (200 mg, 6 ml; UCT), Oasis® HLB (500 mg, 3 ml; Waters) and Strata-X™ (200 mg, 6 ml; Phenomenex). Also, polytetrafluoroethylene (PTFE) syringe filters (13 mm, 0.2 μm; Whatman) were used. The analytical standards of the dyes: malachite green (MG), crystal violet (CV), brilliant green (BG), ethyl violet (EV), methyl violet 2B (MV), pararosaniline (PR), victoria blue B (VBB), victoria blue R (VBR), victoria pure blue BO (VPBBO), methylene blue (MB), azure A (AZA), azure B (AZB), azure C (AZC), thionine (TH), new methylene blue (NMB), nile blue A (NBA), acriflavine (ACR), proflavine (PRO), rhodamine B (RB) and rhodamine 6G (R6G) were all purchased from Sigma-Aldrich (Bornem, Belgium). Malachite green-d_5_ (MG-d_5_) used as an internal standard (IS) was obtained from Witega (Berlin, Germany).

### 2.2 Standard solutions

Individual stock solutions of reference compounds at 1 mg/ml were prepared in acetonitrile (MG, CV, BG, EV, MV, VBB, VBR, VPBBO, NBA, RB, R6G, MG-d_5_), ethanol (TH), methanol (PR) and water (MB, AZA, AZB, AZC, NMB, ACR, PRO), taking into account the content of active substances (stable for at least 3 months). The stock solution of 8 dyes: MG, CV, BG, MV, PR, NMB, RB, R6G were combined and diluted in acetonitrile to prepare working standard solution at concentrations of 100 μg/ml (stable for at least 3 months) [21, 30]. Next, the stock solution of 12 dyes: MB, AZA, AZB, AZC, TH, EV, NBA, ACR, PRO, VBB, VBR, VPBBO were combined and diluted in acetonitrile to prepare a working standard solution at concentrations of 10 μg/ml (stable for at least 3 months) [21]. The working standard solutions of dyes were further diluted with a mixture of ammonium acetate buffer (0.05 M, pH 3.5), acetonitrile and ascorbic acid solution (1 mg/ml) (47.5:47.5:5, v/v/v) to produce working standard solutions at concentrations of 10, 1, 0.1, 0.01, 0.001 μg/ml (stable for at least 1 month) for MG, CV, BG, MV, PR, NMB, RB, R6G and of 1, 0.1, 0.01 μg/ml (stable for at least 1 month) for MB, AZA, AZB, AZC, TH, EV, NBA, ACR, PRO, VBB, VBR, VPBBO.

The stock solution of MG-d_5_ was diluted in acetonitrile to prepare working internal standard solutions of 100 and 10 μg/ml (stable for at least 6 months). The working internal standard solution of MG-d_5_ was diluted with a mixture of ammonium acetate buffer (0.05 M, pH 3.5), acetonitrile and ascorbic acid solution (1 mg/ml) (47.5:47.5:5, v/v/v) to produce a working internal standard solution at concentrations of 1, 0.1 and 0.01 μg/ml (stable for at least 1 month) [21]. Working standard solutions of 8 dyes and 12 dyes were combined with working internal standard solution of MG-d_5_ and further diluted to produce LC-MS/MS calibration standards of 0, 0.25, 1.25, 2.5, 12.5, 25 ng/ml concentrations for MG, CV, BG, MV, PR, NMB, RB, R6G and 0, 2.5, 12.5, 25, 125, 250 ng/ml concentrations for MB, AZA, AZB, AZC, TH, EV, NBA, ACR, PRO, VBB, VBR, VPBBO. The concentration of MG-d_5_ in the calibration standards was 2.5 ng/ml. All standard solutions were prepared in amber volumetric glass flasks and stored at 4 °C.

### 2.3 Water samples

For the method development, river water samples free from pharmacologically active dyes were used as blank samples.

For testing the applicability of the method for determination of 20 pharmacologically active dyes in real samples, different water samples collected in Poland from rivers (5), lakes (5), ponds (5) and WWTP effluents (5) were analysed. The collected water samples (1l) were placed in high-density polyethene (HDPE) bottles, transported to the laboratory on ice and finally stored at - 20 °C.

### 2.4 Quality control and quality assurance

During the analysis of water samples, quality control samples comprising blank and fortified samples were run for each sample series.

### 2.5 Sample preparation

The extraction from water was performed as reported by Mitrowska [21] with some modifications. The water sample was defrosted and then centrifuged at 4000 r/min for 10 min. After that, 50 ml of water from central sample volume was transferred and adjusted to pH 3.5 with glacial acetic acid and MG-d_5_ internal standard fortification solution was added at the concentration level corresponding to 0.1 μg/l. The fortified water sample was transferred into a CLEAN-UP® Diol column previously conditioned with 3 ml of methanol and 3 ml of water. Next, the column was washed with 5 ml of water and dried under vacuum for 10 min. Dye residues were eluted with 2 ml of a mixture containing ammonium acetate buffer (0.05 M, pH 3.5), acetonitrile and ascorbic acid solution in methanol (1 mg/ml) (47.5:47.5:5, v/v/v). The obtained extract was filtered through a PTFE syringe filter and transferred to a vial for the UPLC-MS/MS analysis.

### 2.6 UPLC-MS/MS analysis

All analyses were conducted on UPLC-MS/MS system consisted of an AB Sciex ExionLC UPLC system connected to an AB Sciex API 5500 Qtrap® mass spectrometer controlled by the Analyst® software (version 1.6.3.) (AB Sciex, Foster City, CA, USA). Mass spectrometric analysis was performed with electrospray ionisation (ESI) in positive ion mode. The following MS/MS parameters were used: curtain gas (CUR) - 20, collision gas (CAD) - medium, nebulizer gas (GS1) - 40, heater gas (GS2) - 80, ion spray voltage (IS) - 1500 and temperature (TEM) - 600 °C. Detection was performed in multiple reaction monitoring (MRM) mode. For each dye two transitions and for IS one transition were monitored. Details of the MRM transitions are shown in Table 1. Chromatographic separation was achieved using a Kinetex ® F5 (pentafluorophenyl) core-shell LC column (1.7 μm, 2.1 × 100 mm) and a Security guard ULTRA cartridge UHPLC PFP (pentafluorophenyl) for 2.1 mm ID columns (Phenomenex, Torrance, CA, USA). The temperature in the autosampler was kept at 8 °C and the injection volume was 5 μl. The mobile phase A was 0.05 M ammonium acetate buffer (pH = 3.5) while the mobile phase B was acetonitrile. The elution of the analytes was accomplished with the mobile phase flow of 0.4 ml/min at a temperature of 40 °C with the following gradient elution settings: 0.0–0.2 min 10%, from 0.3-3.5 min 90%, 3.6–5.0 min 10% of the mobile phase B.

**Table 1:** UPLC-MS/MS parameters used for the quantitation and the confirmation of 20 pharmacologically active dyes in water.

Analyte	Retention time (min)	Precursor ion (m/z)	Product ion (m/z)	DP (V)	CE (V)	CXP (V)	Dwell time (ms)
malachite green (MG)	2.14	329.3	313.2 208.4	50 50	51 46	20 12	20
crystal violet (CV)	2.34	372.2	356.2 340.5	50 50	54 71	21 19	20
brilliant green (BG)	2.68	385.5	341.3 297.1	50 50	52 72	21 17	15
ethyl violet (EV)	3.33	456.8	412.3 368.3	46 46	62 81	20 20	15
methyl violet 2B (MV)	2.09	358.5	342.3 326.4	44 44	52 71	22 20	20
pararosaniline (PR)	1.55	288.2	194.8 168.1	57 57	44 54	18 9	20
victoria blue B (VBB)	2.33	470.3	454.1 349.2	100 100	58 52	12 22	5
victoria blue R (VBR)	2.32	422.3	406.2 393.2	110 110	51 54	10 23	20
victoria pure blue BO (VPBBO)	2.85	478.2	434.4 390.3	115 115	59 76	10 9	20
methylene blue (MB)	1.67	284.2	268.2 252.2	35 35	48 71	15 15	20
azure A (AZA)	1.55	256.2	213.8 199.1	38 38	46 58	12 11	20
azure B (AZB)	1.60	270.0	254.1 212.1	72 72	47 67	15 14	20
azure C (AZC)	1.50	242.1	199.9 227.0	34 34	47 38	12 12	15
thionine (TH)	1.45	228.2	195.9 210.8	33 33	41 42	10 12	20
new methylene blue (NMB)	1.87	312.3	283.2 254.0	82 82	41 51	13 15	20
nile blue A (NBA)	1.89	318.3	274.0 260.0	74 74	47 71	16 13	15
acriflavine (ACR)	1.50	224.2	208.9 181.9	35 35	38 49	12 17	20
proflavine (PRO)	1.48	210.1	192.9 165.8	25 25	39 46	11 9	20
rhodamine B (RB)	1.88	443.2	399.1 355.1	90 90	58 80	12 12	5
rhodamine 6G (R6G)	2.19	442.2	415.3 341.1	105 105	47 65	12 12	20
malachite green- d_5_ (MG-d_5_)	2.13	334.4	318.2	50	51	19	20

### 2.7 Method validation

The developed method was validated in terms of linearity, specificity, precision, intermediate precision, recovery, limit of detection (LOD) and limit of quantification (LOQ). Quantification was based on peak area and was performed using internal standard solvent-based calibration curve with 1/x weighting obtained from analysing LC-MS/MS calibration standards. To check linearity matrix-matched calibration samples in the range from 0 to 5 μg/l (0, 0.005, 0.01, 0.05, 0.1, 0.5, 1, 5 μg/l) were analysed on three separate days. The specificity of the method was verified by analysis of 20 different water samples from rivers, lakes, ponds and WWTP effluents. To evaluate precision, 6 blank deionised water samples were fortified with MG, CV, BG, MV, PR, NMB, RB, R6G at three different concentration levels: 0.01, 0.05, 0.1 μg/l and with MB, AZA, AZB, AZC, TH, EV, NBA, ACR, PRO, VBB, VBR, VPBBO at 0.1, 0.5 and 1 μg/l. The prepared samples were analysed on the same day at the same instrument and by the same operator and relative standard deviation (RSD, %) was calculated for each fortification level. This was repeated on another two days with the same instrument but by different operators and intermediate precision was evaluated by calculating RSD (%) on three separate occasions. Recovery (%) was calculated by comparing the obtained mean measured concentration with the fortified concentration of the tested samples. To calculate LOD, a signal-to-noise (S/N) of at least three was used and LOQ was defined as S/N of at least ten.

### 2.8 Matrix effect

To assess the matrix effect, the peak areas of individual analytes obtained after fortification of the extract (B) were compared to the area of the analytes peaks obtained in the standard solutions (A) using the Eq. (1).

(1) ME (%) = B/A × 100

ME = 100 % – matrix effect not observed;

ME > 100% – matrix effect causes ionisation enhancement;

ME < 100% – matrix effect causes ionisation suppression [31].

### 2.9 Statistical analysis

All samples were carried out in three replicates and data were expressed as means ± standard deviations. Analysis of variance (ANOVA) was used to test the significance of differences.

## 3 Results and discussion

### 3.1 UPLC-MS/MS optimisation

#### 3.1.1 MS/MS optimisation

Tandem mass spectrometry analyses were performed with ESI in positive ion mode. The mass spectrometer compound-dependent parameters were optimised by direct infusion of a standard solution of each tested analyte 10 ng/ml in a mixture of ammonium acetate buffer (0.05 M, pH 3.5), acetonitrile and ascorbic acid solution (1 mg/ml) (47.5:47.5:5, v/v/v) from syringe pump at the rate of 10 μl/min. The presence of each tested compound was confirmed by Multi-Channel Analysis (MCA) scans and next, pre-collision cell voltages including declustering potential (DP), entrance potential (EP) and collision cell entrance potential (CEP) were optimised. The expected molecular ion with maximum signal intensity was selected as a precursor ion. The positively charged ions [M]^+^ were recorded for all of the selected dyes. After fragmentation of the selected precursor ion, two most intense product ions were selected. For each product ion parameters such as collision energy (CE) and collision cell exit potential (CXP) were optimised. Finally, MRM mode was used and for each dye two MRM transitions were selected; primary transition as quantifier and secondary one as qualifier while for IS one MRM transition was chosen (Table 1). Next, ion source parameters including curtain gas (CUR), collision gas (CAD), ion source gases: nebulizer gas (GS1) and heater gas (GS2), ion spray voltage (IS) and temperature (TEM) were optimised by Flow Injection Analysis (FIA) using a standard solution containing a mixture of 20 dyes at concentration of 10 ng/ml. Each ion source parameter: CUR (10, 20, 30, 40, 50), CAD (low, medium, high), GS1 (20, 40, 60, 80), GS2 (20, 40, 60, 80), IS (500, 1000, 1500, 2000, 2500, 3000, 3500, 4000, 4500, 5000) and TEM (100, 200, 300, 400, 500, 600) was optimised in triplicate. The results of ion source optimisation for individual dye compounds and for each tested class of dyes are presented in Figure S1 and Figure S2, respectively (Supplemental Information). The final value of selected parameters was based on the highest mean signal intensity at tested settings and was as follows: CUR = 20, CAD = medium, GS1 = 40, GS2 = 80, IS = 1500 and TEM = 600 °C. The results showed that changes in values of ion source parameters such as IS, GS2 and TEM have the most important impact on signal intensity of tested dyes. The most susceptible for changes of IS and GS2 were phenothiazine, triphenylmethane and xanthene dyes. On the other hand, interface temperature impacted on signal intensity of all tested dyes. Thus, additional tests were conducted to optimise the most susceptible ion source parameters (IS, GS2, TEM) (Figure S3, Table S1). It turned out that only a change of ion spray voltage from 1000 V to 1500 V resulted in higher signal intensity for triphenylmethane and xanthene dyes (SET5). Unfortunately, for phenothiazine dyes IS above 1000 V leads to lower signal intensity of the dyes for which this value was the most suitable (SET3). On the other hand, increased IS (1500 V) in a combination with higher TEM (600 °C) (SET8) presents the best ion source conditions for triphenylmethane and xanthene dyes signal intensity (Figure S3, Table S1). Finally, initially selected ion source settings: CUR = 20, CAD = medium, GS1 = 40, GS2 = 80, IS = 1500 and TEM = 600 °C were confirmed (Table S2).

#### 3.1.2 UPLC optimisation

The impact of mobile phases, type of analytical columns and column temperature on the separation of 20 dyes was tested. The optimisation of chromatographic separation was performed by injection of a mixture of 20 dyes at a concentration of 10 ng/ml. Different reversed phase analytical columns such as Kinetex ® C18 (100 × 2.1 mm id, 1.7 μm), Kinetex ® F5 (100 mm × 2.1 ​mm id, 1.7 μm) with ammonium acetate buffer (0.05 ​M, pH = 3.5) and acetonitrile as mobile phases were tested. Also, types of elution (isocratic, gradient), as well as different programs of elution, were tested. The best chromatographic separation of all analytes in terms of peak width, symmetry and time of analysis was achieved on Kinetex ® F5 column in a gradient elution and thus it was selected for further UPLC optimisation steps. The F5 stationary phase is pentafluorophenyl phase with TMS (trimethyl silane) endcapping combined with core-shell silica that offers a combination of polar, hydrophobic, aromatic and shape selectivity and allows high analytes retention, sensitivity and reproducibility. So far, no method using a pentafluorophenyl (F5) analytical column for the separation of pharmacologically active dyes in water has been described in the literature. The only known method of separation of dyes on this chromatographic column concerns the determination of malachite green and leucomalachite green in bottom sediments [32].

**Figure 2:**
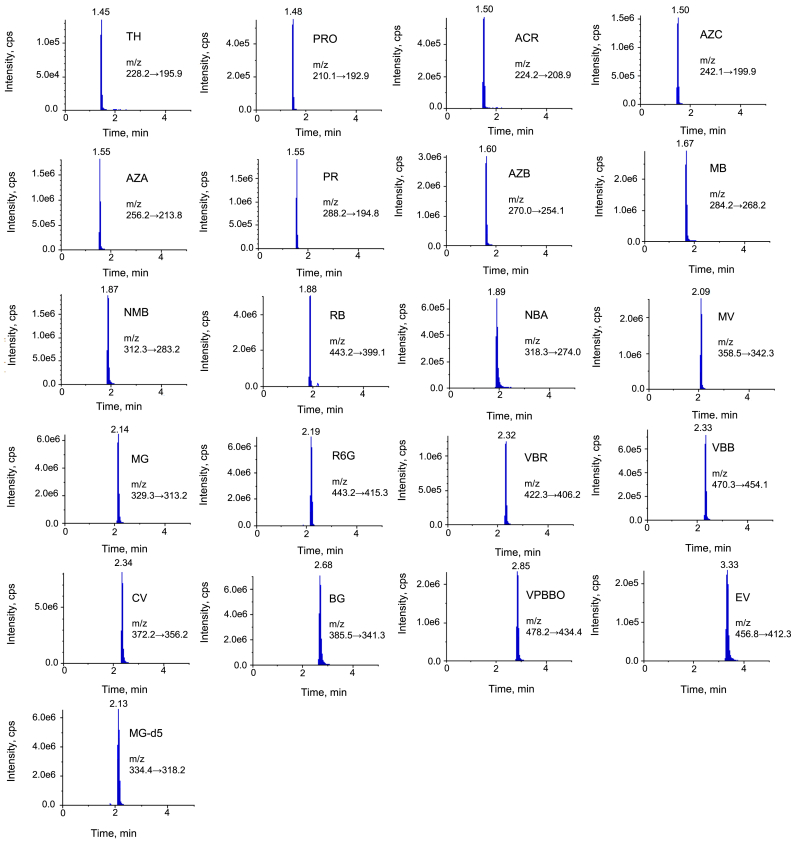
Chromatographic separation of 20 pharmacologically active dyes in water at concentration level of 0.1 μg/l for MG, CV, BG, MV, PR, NMB, RB, R6G and 1.0 μg/l for EV, VBB, VBR, VPBBO, MB, AZA, AZB, AZC, TH, NBA, ACR, PRO and an internal standard (MG-d5).

Next, mobile phases including 0.05 M ammonium acetate buffer (pH = 3.5), 0.05 M ammonium formate buffer (pH = 3.5), acetonitrile were tested. The application of 0.05 M ammonium acetate buffer (pH = 3.5), and acetonitrile as mobile phases allows obtaining improved peaks shape and signal intensity for phenothiazine dyes than the use of 0.05 M ammonium formate buffer (pH = 3.5) and acetonitrile. Sample extract injected to UPLC-MS/MS consisted of a mixture of ammonium acetate buffer (0.05 M, pH 3.5), acetonitrile and ascorbic acid solution (1 mg/ml) (47.5:47.5:5, v/v/v). To keep the organic solvents compatible, acetonitrile was chosen as the organic modifier in the mobile phase. Finally, mobile phases of 0.05 M ammonium acetate buffer (pH = 3.5) and acetonitrile were selected because their application allowed obtaining chromatographic separation of all tested dyes since the degree of the pH of buffer solution is an important factor in the chromatographic separation of tested dyes which degree of ionisation depends on the pH of the mobile phase. Based on pKa values calculated with Chemicalize platform [33], most of the tested dyes are acidic compounds with pKa ranging from 2.1 to 6.87 except for MV, PR, PRO and NBA which are weak basic compounds with pKa range from 8.32 to 10.29 (Figure 1). When pKa value of a compound is higher than pH, it is in an ionic state. Thus, when pH of a solution increase, a decreased recovery is observed. Therefore, the range of pH of ammonium acetate buffer from 3 to 6 was also verified. In addition, column temperature (from 25 °C to 50 °C), injection volume (from 3 μl to 10 μl) and flow rate (from 0.2 ml/min to 0.5 ml/min) were tested. As a result of optimisation, the final optimised UPLC settings included 0.05 M ammonium acetate buffer solution with pH adjusted to 3.5 and acetonitrile as mobile phases, column temperature of 40 °C, sample injection volume of 5 μl and 0.4 ml/min of flow rate which allowed separating all 20 dyes within 5 min (Table S3, Figure 2). The obtained total time of chromatographic separation (5 min) is shorter in comparison to other methods which allows determining up to 3 pharmacologically active dyes in water: 8 min [7], 15 min [21], 16 min [8].

### 3.2 Sample preparation optimisation

Based on the chemical structure of tested compounds and literature data three types of SPE sorbents: silica normal diol phase (BAKERBOND spe™ Diol and CLEAN-UP® Diol columns), polymeric hydrophilic-lipophilic-balanced reversed phase (Oasis® HLB columns) and polymeric reversed phase (Strata-X™ columns) were selected to test the recovery of 20 dyes. The tested SPE columns were used according to protocols supplied by manufacturers considering factors and conditions important for their effectiveness. To verify SPE columns, the deionised water samples (pH = 3.5) were fortified with a mixture of dyes at a concentration of 1 μg/ml.

The results showed that application of BAKERBOND spe™ Diol and CLEAN-UP® Diol columns allowed recovering all tested dyes. In the case of Oasis® HLB columns, 17 out of 20 dyes were extracted except for EV, VBB and VPBBO while Strata-X™ columns allowed the extraction of only 9 dyes (MB, AZA, AZB, AZC, TH, NM, ACR, PRO, PR). The highest recovery range of dyes was obtained with CLEAN-UP® Diol (from 58.77 % to 95.75 %) and BAKERBOND spe™ Diol (from 31.4 % to 86.75 %) whereas for other SPE columns, which did not allow recovering all tested dyes, the recovery range was from 15.33 % to 94.4 % for Oasis® HLB columns and from 1.08 % to 12.73 % for Strata-X™ columns (Figure S4). In comparison to BAKERBOND spe™ Diol dyes recoveries using CLEAN-UP® Diol were higher for all 20 dyes except for RB. The highest recovery difference achieved for CLEAN-UP® Diol in contrast to BAKERBOND spe™ Diol were for: PRO (31.3 %), AZB (30.77 %), ACR (28.98 %), TH (28.83 %), AZA (28.05 %), PR (27.45 %), MB (26.93 %), AZC (24.07 %), NMB (22.37 %), CV (21.35 %) and BG (21.35 %). Application of BAKERBOND spe™ Diol allowed obtaining higher recovery only for RB (80.27 %) in comparison to CLEAN-UP® Diol (77.98 %). Therefore, considering obtained recovery results for different tested columns (three replicates), CLEAN-UP® Diol columns were finally selected for the procedure of 20 pharmacologically active dyes extraction from aquatic samples.

The pH of aquatic solution plays a crucial role in dyes ionisation and it should be considered during analytical method optimisation. The pKa values (2.34–10.29) (Figure 1) of the functional groups associated with tested dyes have an impact on their charge state which directly depends on the pH of a solution. Thus, an impact of pH (from 2 to 12) of water sample fortified with a mixture of 20 dyes at concentration of 1 μg/mL on the recovery using selected CLEAN-UP® Diol columns was tested.

**Figure 3:**
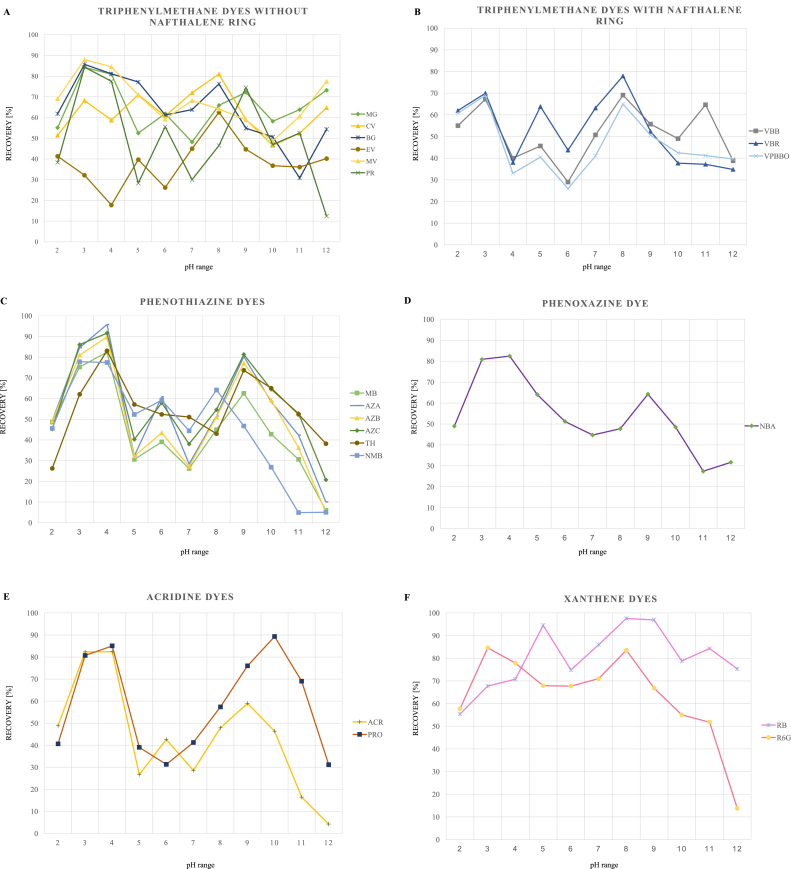
Mean percent recoveries of 20 pharmacologically active dyes from chemical class of (A) triphenylmethane without naphthalene ring, (B) triphenylmethane without naphthalene ring, (C) phenothazine, (D) phenoxazine, (E) acridine, (F) xanthene dyes obtained from water samples of various pH values (n = 3).

Tests indicated that obtained recoveries of tested dyes strongly depended on pH values of aquatic solution (Figure 3). For triphenylmethane dyes without naphthalene ring (except for CV and EV) the highest recoveries (three replicates), were obtained at pH = 3 but for triphenylmethane dyes with naphthalene ring: VBB, VBR, VPBBO (Figure 1) pH = 8 allows obtaining the highest recoveries. The highest recoveries for phenothiazine and phenoxazine dyes were obtained at pH = 4 and for xanthene dyes depending on a dye at pH = 3 (for RG6) and at pH = 8 (for RB).

For most of the tested dyes, the highest recovery was obtained with pH = 3 (for MG, BG, MV, PR, ACR, R6G) and pH = 4 (for MB, AZA, AZB, AZC, TH, NMB, NBA). As a result, pH = 3.5 was selected; for other dyes, pH = 3.5 allowed achieving acceptable recoveries (>70 %). The value of pH = 3.5 is close to other authors’ results where pH of 3 was used for the determination of triphenylmethane [19, 21] and xanthene [8, 24] dyes and pH of 4 for determination of triphenylmethane [7, 17] in water samples.

### 3.3 Method validation

The developed method was validated in terms of linearity, specificity, precision, recovery, LOD and LOQ. Due to the use of the isotopically labelled compound (MG-d_5_) as an internal standard, it was not necessary to use a matrix-matched calibration curve. The concentrations of the analytes in the samples were calculated by solvent-based calibration curve using an internal standard. Calibration curves with 1/x weighting obtained from analysing LC-MS/MS calibration standards were plotted for each individual analyte. The solvent-matched calibration curves were linear over the range 0.25–25 ng/ml with coefficient of determination (R^2^) above 0.995 for MG, CV, BG, MV, PR, NMB, RB, R6G and over the range 2.5–125 ng/ml with R^2^ above 0.998 for MB, AZA, AZB, AZC, TH, EV, NBA, ACR, PRO, VBB, VBR, VPBBO (Table S4). The calibration curves generated from matrix-matched calibration curves were linear (R^2^ > 0.994) over the range 0.01–5 μg/l for MG, CV, BG, MV, PR, NMB, RB, R6G and 0.1–5.0 μg/l for MB, AZA, AZB, AZC, TH, EV, NBA, ACR, PRO, VBB, VBR, VPBBO (R^2^ > 0.996) The specificity was evaluated by the analysis of 20 different blank environmental water samples (river, lake, pond, WWTP effluent) (Figure S5). As a result, no interfering peaks were found in the retention time of each of the analytes confirming the specificity of the developed method. The obtained recovery values for all tested analytes were between 71.2 and 104.9% with a good RSD, less than 14 % at all fortification levels (0.01, 0.05, 0.1 μg/l and 0.1, 0.5, 1.0 μg/l; Table 2). Although the LOQ was calculated with S/N (≥10) it was verified in practice by fortifying water sample at LOQ level and finally was set at the lowest value of the matrix-matched calibration curve in the linear range. The method was the most sensitive for MG, CV, BG, MV, PR, NMB, RB, R6G, for which LOD and LOQ was 0.003 μg/l and 0.01 μg/l, respectively. LOD and LOQ for MB, AZA, AZB, AZC, TH, EV, NBA, ACR, PRO, VBB, VBR and VPBBO was 0.03 μg/l and 0.1 μg/l, respectively (Table 2, Figure 2). The validation results have shown that the proposed method is sensitive, specific and accurate in determining all selected dyes in different water samples (river, lake, pond, WWTP effluent). The estimated matrix effect for the 20 dyes showed values ranging from 70.5 to 93.5% indicating ionisation suppression (ME <100%).

**Table 2:** Validation results for the UPLC-MS/MS method for the determination of 20 pharmacologically active dyes in environmental water.

Analyte	LOD (μg/l)	LOQ (μg/l)	Concentration level (μg/l)	Recovery (%)	Precision (RSD, %)	Intermediate precision (RSD, %)
MG	0.003	0.01	0.01	102.1	5.4	9.8
			0.05	100.6	4.8	8.6
			0.1	101.6	4.4	8.2
CV	0.003	0.01	0.01	99.3	3.9	6.9
			0.05	100.0	4.8	7.6
			0.1	103.1	6.0	8.5
BG	0.003	0.01	0.01	98.7	7.8	9.2
			0.05	99.2	7.5	9.8
			0.1	98.4	8.4	10.3
EV	0.03	0.1	0.1	98.7	12.8	13.2
			0.5	99.8	11.5	12.7
			1.0	103.4	10.3	11.6
MV	0.003	0.01	0.01	99.8	6.5	7.3
			0.05	101.4	6.8	7.9
			0.1	102.3	5.2	7.4
PR	0.003	0.01	0.01	73.5	6.5	10.5
			0.05	71.2	5.4	9.8
			0.1	75.4	5.0	9.6
VVB	0.03	0.1	0.1	102.1	7.4	9.5
			0.5	104.9	8.1	10.3
			1.0	103.7	7.0	9.9
VBR	0.03	0.1	0.1	98.3	4.7	6.8
			0.5	96.3	5.6	7.0
			1.0	95.5	6.0	7.5
VPBBO	0.03	0.1	0.1	96.0	7.8	10.5
			0.5	102.7	7.0	9.1
			1.0	101.7	6.9	9.5
MB	0.03	0.1	0.1	97.5	6.7	8.9
			0.5	95.5	8.5	9.7
			1.0	102.8	9.0	10.5
AZA	0.03	0.1	0.1	101.1	10.0	11.5
			0.5	100.0	8.8	10.8
			1.0	99.3	7.9	10.2
AZB	0.03	0.1	0.1	97.2	8.8	11.0
			0.5	100.2	9.5	11.7
			1.0	98.6	8.0	10.7
AZC	0.03	0.1	0.1	97.3	6.7	8.9
			0.5	102.2	7.6	9.7
			1.0	95.3	8.9	10.2
NMB	0.003	0.01	0.01	99.8	10.0	11.4
			0.05	89.9	8.9	10.7
			0.1	90.9	9.7	11.0
TH	0.03	0.1	0.1	102.3	7.9	10.1
			0.5	100.1	8.5	11.5
			1.0	99.1	9.9	12.2
NBA	0.03	0.1	0.1	100.3	7.6	8.9
			0.5	94.8	7.1	8.1
			1.0	97.8	8.6	10.1
ACR	0.03	0.1	0.1	100.2	7.9	9.2
			0.5	98.9	6.5	8.3
			1.0	100.1	6.9	9.9
PRO	0.03	0.1	0.1	99.9	6.5	8.1
			0.5	96.7	5.4	7.6
			1.0	103.2	5.1	7.4
RB	0.003	0.01	0.01	102.3	7.8	9.9
			0.05	100.4	8.9	10.2
			0.1	97.6	9.1	10.7
R6G	0.003	0.01	0.01	95.4	7.4	8.8
			0.05	98.9	7.0	8.3
			0.1	97.2	6.8	7.8

### 3.4 Application to real samples

The proposed method was successfully applied to detect and quantify 20 pharmacologically active dyes in the water samples collected from rivers (5), lakes (5), ponds (5) and WWTP effluents (5). The results showed that among tested water samples dyes were found only in two WWTP effluent samples. In one of the samples only RB was determined (0.043 μg/l) and the other one contained CV (0.023 μg/l), MV (0.017 μg/l) and RB (0.027 μg/l). Thus, the method can be used to assess the exposure of water samples to 20 pharmacologically active dyes.

### 3.5 Comparison of the developed method with other approaches for determination of dyes in water samples

It is worth emphasising that this is the first multi-compound method that allows in one simple analytical approach the detection of 20 pharmacologically active dyes belonging to 5 different chemical classes in water samples at low concentration (LOD = 0.003 μg/l).

So far, published methods enable to detect individual dye [17, 22, 24], two dyes [7, 8, 21, 28, 34] or up to three pharmacologically active dyes [23, 29] in water samples. The developed method allows the detection and quantification of a wider range of pharmacologically active dyes (20) than other available methods. Additionally, the presented method enables, for the first time, to determine pharmacologically active dyes such as ACR, PRO, AZA, AZC, TH, NMB, NBA, EV, PR, VBB, VBR, VPBBO in water samples.

Until now there have been a few published methods for determination of dyes in water samples allowing the detection and quantification of dyes at the concentration level below 1 μg/l. LOQ for MG (0.01 μg/l) in the presented method is lower than LOQ obtained with methods developed by Mitrowska [21] (0.04 μg/l), Khan [12] (0.1 μg//l), Zhang [7] (0.25 μg/l), Fang [35] (0.5 μg/l) and Ghasemi [23] (13.6 μg/l). LOQ of another triphenylmethane dye, CV (0.01 μg/l) allows the determination of the dye at lower concentration than LOQ obtained with other authors' methods: Zhang [7] (0.25 μg/l), Šafařík [34] (0.5 μg/l), Sadeghi [27] (4.7 μg/l), An [19] (16 μg/l), Ghasemi [23] (17.6 μg/l), Aydin [20], Yu [16] (32 μg/l). LOQ obtained for BG (0.01 μg/l) is also significantly lower than LOQ presented in other papers: Es'haghi [17] (1.83 μg/l), Sadeghi [27] (9 μg/l) and Hosseini [36] (40 μg/l).

The published methods allow the determination of MB in wastewater [13] and stream water [29] with LOQ equal to 200 μg/l and 150 μg/l, respectively. The presented method allows quantifying MB in the aquatic samples at 0.1 μg/l concentration level. Up to now, there has been developed only one method which allows detecting another phenothiazine dye, AZB in water samples [28] with LOQ (5.72 μg/l) higher than LOQ in the proposed method (0.1 μg/l).

Xanthene dyes, such as RB and R6G, have been determined in river water and WWTP effluents with LOQ equal to 2 μg/l and 0.5 μg/l, respectively [8]. Also, Biparva [15] developed a method for the determination of R6G in wastewater with LOQ = 7.97 μg/l. The available methods have definitely higher LOQ than those obtained in the developed method (0.01 μg/l).

## 4 Conclusions

This is the first multi-compound method that allows concomitant determination of 20 pharmacologically active dyes in different water samples from rivers, lakes, ponds and WWTP effluents. The developed method allowed the determination for the first time of pharmacologically active dyes such as ACR, PRO, AZA, AZC, TH, NMB, NBA, EV, PR, VBB, VBR, VPBBO in water samples.

The developed method is based on clean-up on diol SPE cartridges followed by UPLC-MS/MS that allows achieving LOQs as low as 0.01–0.1 μg/l (depending on the dye) that is sensitive enough to quantify very low concentration levels of the dyes in water sample. The method has been successfully validated and proved to be accurate and precise for the determination of all tested compounds. Additionally, the advantage of the presented method is the short total time of chromatographic separation of 20 pharmacologically active dyes achieved within 5 min by using a pentafluorophenyl (F5) analytical column and mobile phases of ammonium acetate buffer (0.05 M, pH = 3.5) and acetonitrile with gradient elution.

Application of the proposed method to real samples allows the determination of CV, MV and RB in the WWTP effluents. This confirms that dyes pose an environmental problem and a developed credible method was needed. As indicated, the proposed method can be used in the control, research and risk assessment of the pharmacologically active dyes occurrence in the aquatic environment.

## Declarations

### Author contribution statement

Angelika Tkaczyk-Wlizło: Conceived and designed the experiments; Performed the experiments; Analyzed and interpreted the data; Wrote the paper.

Kamila Mitrowska: Conceived and designed the experiments; Analyzed and interpreted the data; Contributed reagents, materials, analysis tools or data; Wrote the paper.

Tomasz Błądek: Performed the experiments; Analyzed and interpreted the data.

### Funding statement

This work was supported by 10.13039/501100004569Ministerstwo Nauki i Szkolnictwa Wyższego (05-1/KNOW2/2015).

### Data availability statement

Data included in article/supplementary material/referenced in article.

### Declaration of interests statement

The authors declare no conflict of interest.

### Additional information

No additional information is available for this paper.
