# Supplementary material for: Quantification of twenty pharmacologically active dyes in water samples using UPLC-MS/MS
Source: Heliyon. 2022 Apr 23;8(4):e09331. doi: 10.1016/j.heliyon.2022.e09331 (PMC9062210; doi:10.1016/j.heliyon.2022.e09331)
Supplement: Supplemental Information [file mmc1.docx]

**Supplemental Information**

Supplemental items:

**Figure S1.** The results of optimisation of ion source parameters: (A) curtain gas, (B) collision gas, (C) nebulizer gas, (D) heater gas, (E), (F) ion spray voltage, and (G) interface temperature achieved for 20 pharmacologically active dyes (n= 3).

**Figure S2.** The results of optimisation of ion source parameters: (A) curtain gas, (B) collision gas, (C) nebulizer gas, (D) heater gas, (E) ion spray voltage, (F) interface temperature achieved for individual chemical classes: acridine, phenothazine, phenoxazine, tripenylmethane and xanthene pharmacologically active dyes (n= 3).

**Figure S3.** The results from additional tests (SET1-9) of optimisation of selected ion source parameters (Table S1) achieved for individual chemical classes: acridine, phenothazine, phenoxazine, tripenylmethane and xanthene pharmacologically active dyes (n= 3).

**Figure S4.** Mean percent recoveries of 20 pharmacologically active dyes from water samples using various SPE columns (n= 3).

**Figure S5.** Multiple reaction monitoring (MRM) chromatograms of 20 pharmacologically active dyes: TH, PRO, ACR, AZC, AZA, PR, AZB, MB, NMB, RB, NBA, MV, MG, R6G, VBR, VBB, CV, BG, VPBBO, EV and an internal standard (MG-d5) in blank water sample.

**Table S1.** Selected parameters of heater gas (GS2), ion spray voltage (IS), interface temperature (TEM) used in additional tests (SET1-9) during ion source optimisation.

**Table S2.** Selected values for ion source parameters used for tandem mass spectrometry determination of 20 pharmacologically active dyes.

**Table S3.** Selected values for chromatographic separation of 20 pharmacologically active dyes.

**Table S4.** Regression equations and coefficients of determination (R^2^) used in the method for determination of 20 pharmacological active dyes in water.

**Abbreviations of dyes:**

ACR, acriflavine; AZA, azure A; AZB, azure B; AZC, azure C; BG, brilliant green; CV, crystal violet; EV, ethyl violet; MB, methylene blue; MG, malachite green; MV, methyl violet 2B; NBA, nile blue A; NMB, new methylene blue; PR, pararosaniline; PRO, proflavine; RB, rhodamine B; R6G, rhodamine 6G; TH, thionine; VBB, victoria blue B; VBR, victoria blue R; VPBBO, victoria pure blue BO.

**A**


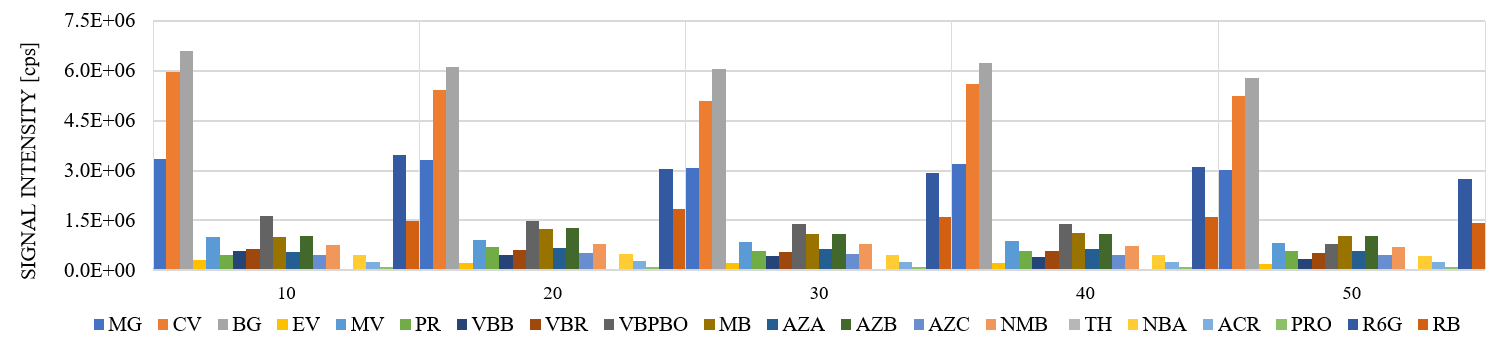


**CURTAIN GAS**

**B**


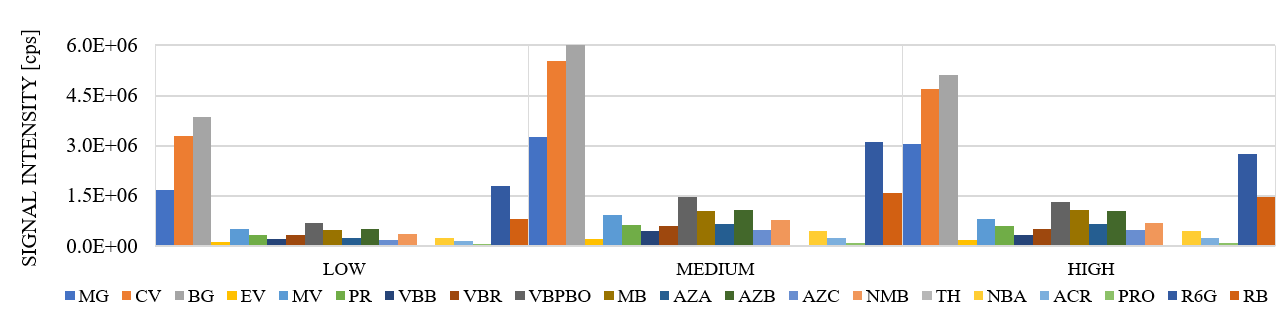


**COLLISION GAS**

**C**


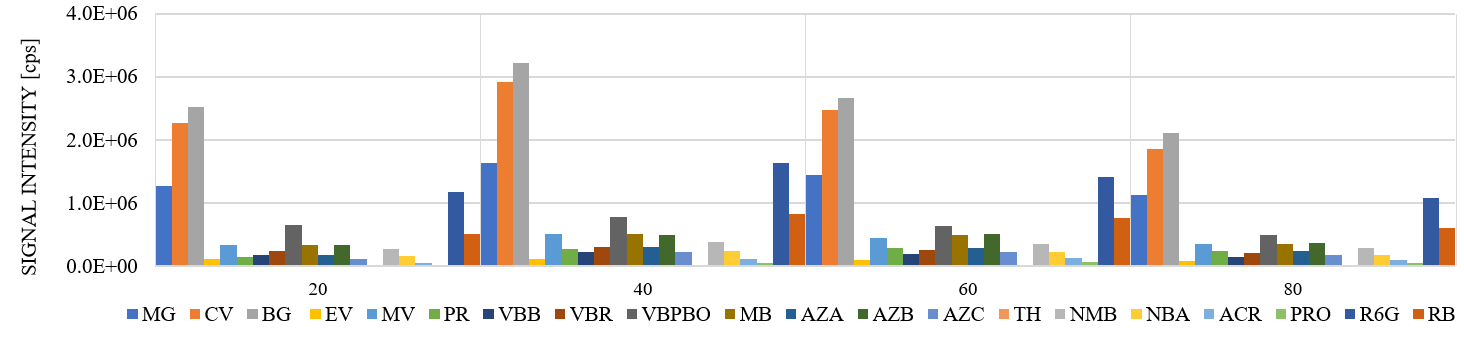


**NEBULIZER GAS (PSI)**

**D**


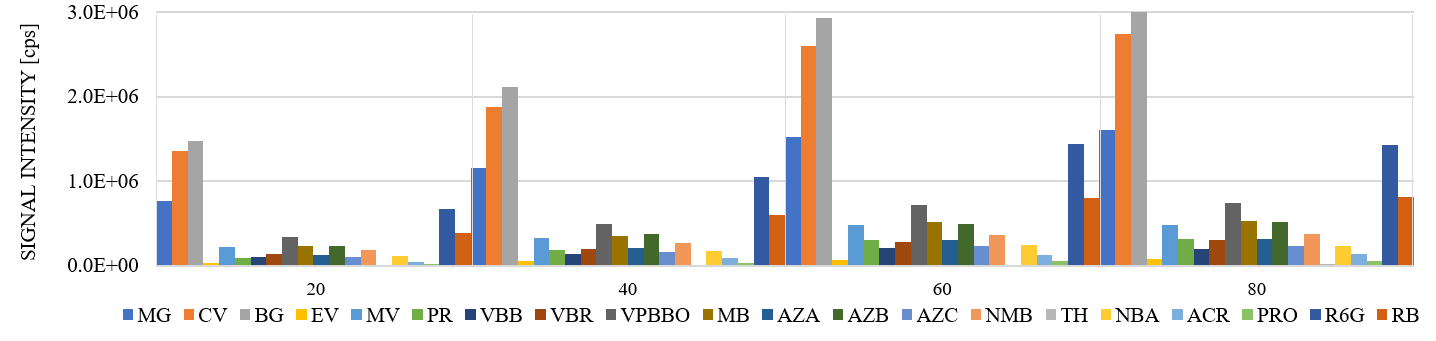


**HEATER GAS (PSI)**

**E**


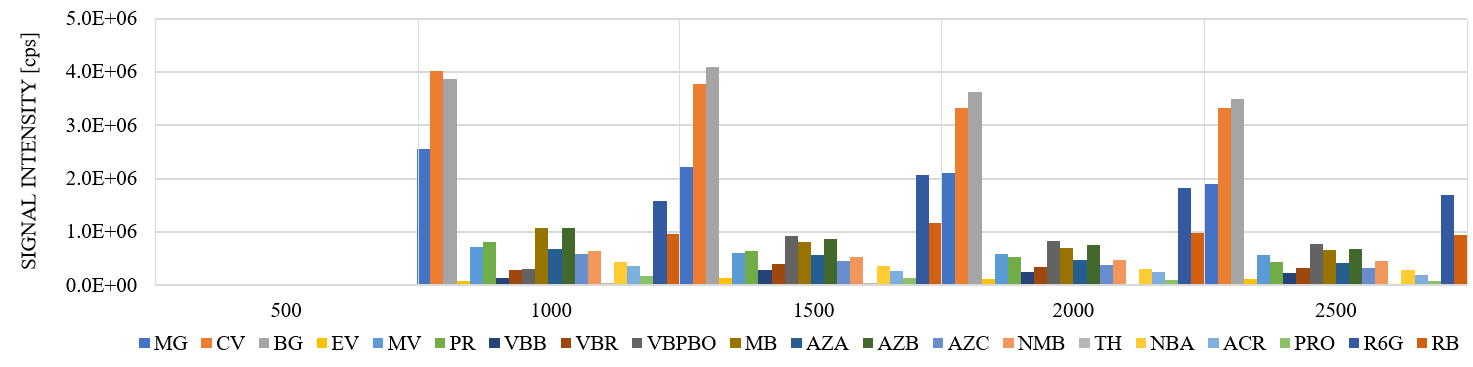


**ION SPRAY VOLTAGE (V)**

**F**


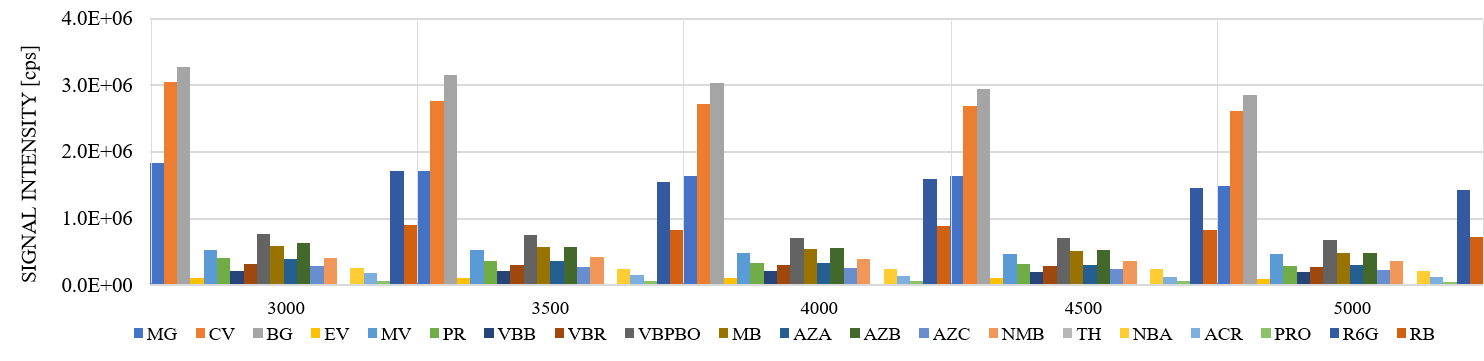


**ION SPRAY VOLTAGE (V)**

**G**


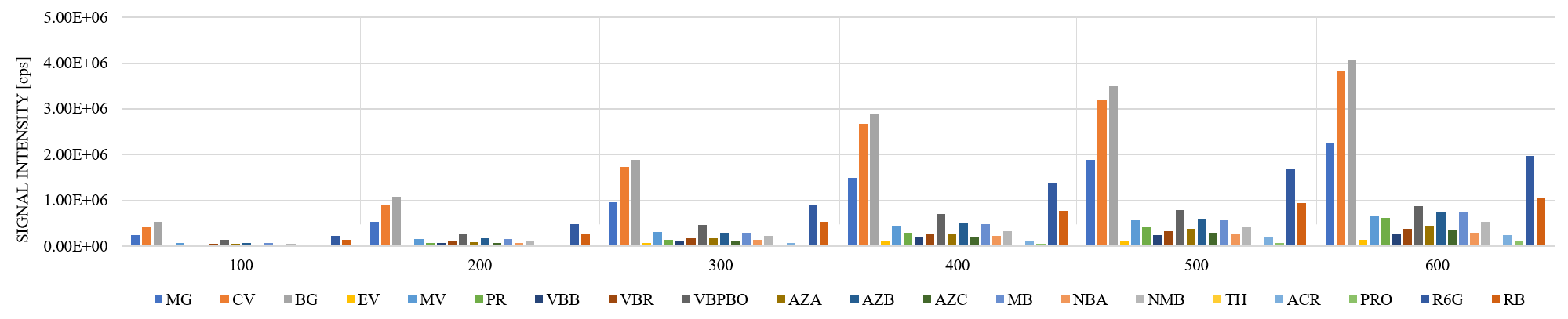


**INTERFACE TEMPERATURE (°C)**

**Figure S1.** The results of optimisation of ion source parameters: (A) curtain gas, (B) collision gas, (C) nebulizer gas, (D) heater gas, (E, F) ion spray voltage, and (G) interface temperature achieved for 20 pharmacologically active dyes (n= 3).

**Figure S2.** The results of optimisation of ion source parameters: (A) curtain gas, (B) collision gas, (C) nebulizer gas, (D) heater gas, (E) ion spray voltage, (F) interface temperature achieved for individual chemical classes: acridine, phenothazine, phenoxazine, tripenylmethane and xanthene pharmacologically active dyes (n= 3).


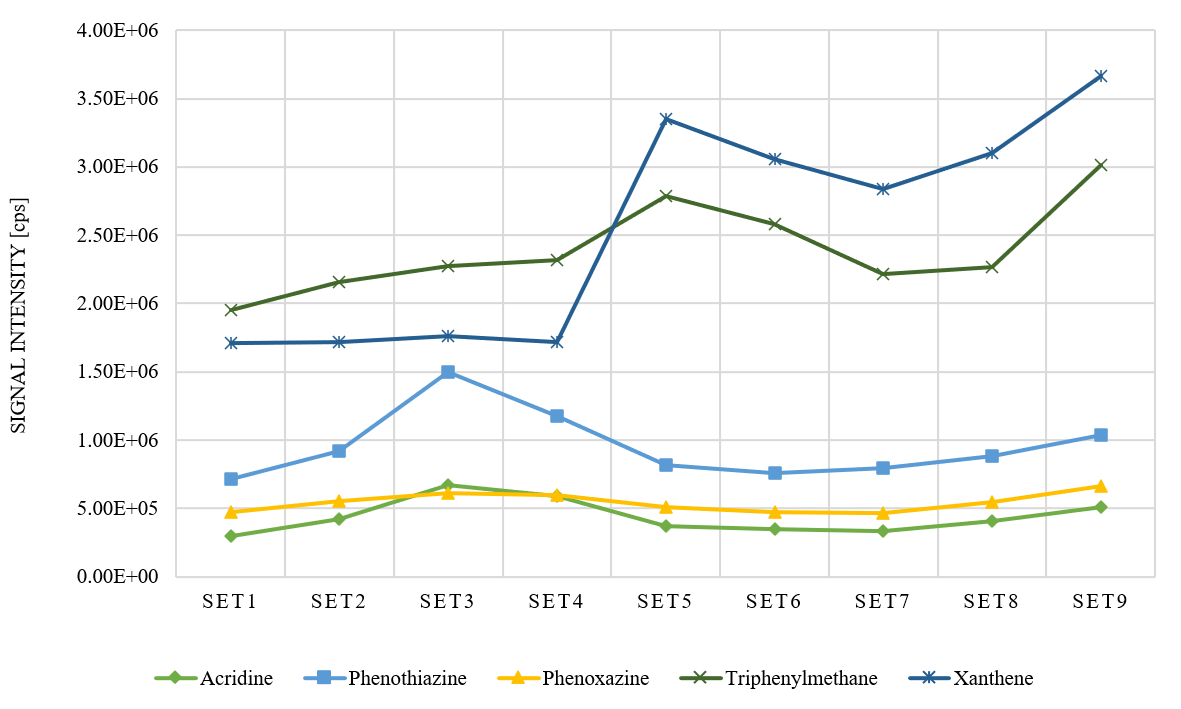


**Figure S3.** The results from additional tests (SET1-9) of optimisation of selected ion source parameters (Table S1) achieved for individual chemical classes: acridine, phenothazine, phenoxazine, tripenylmethane and xanthene pharmacologically active dyes (n= 3).


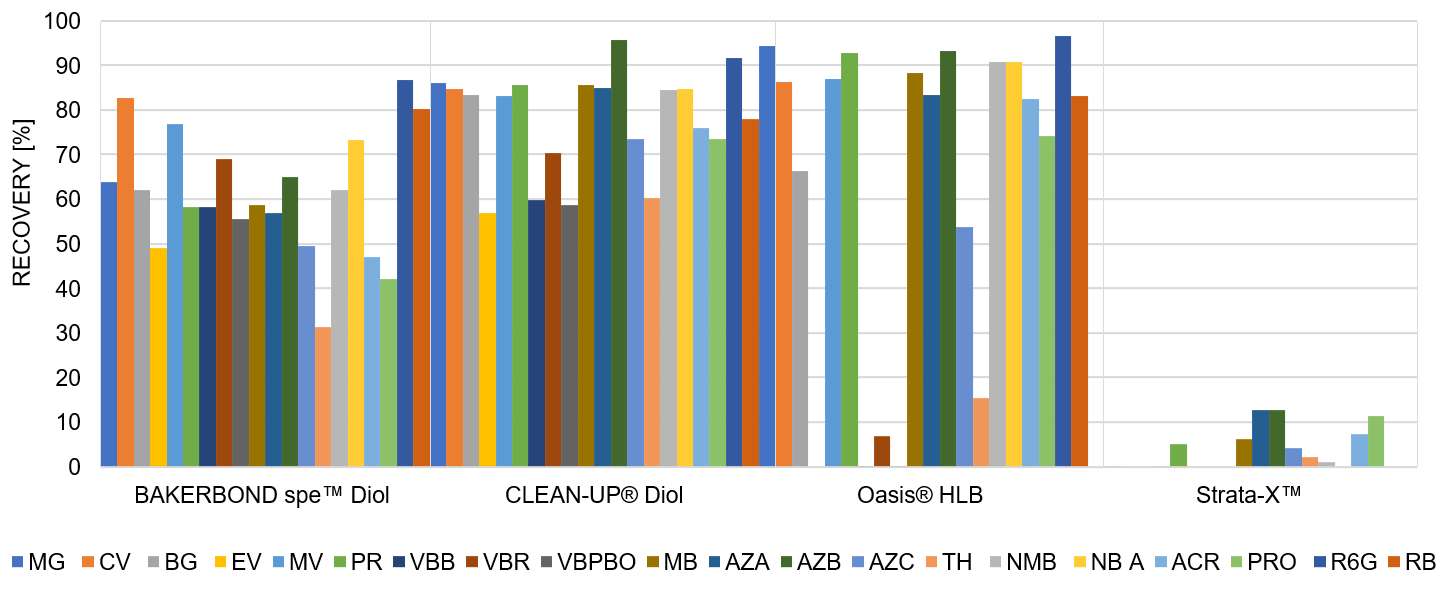


**Figure S4.** Mean percent recoveries of 20 pharmacologically active dyes from water samples using various SPE columns (n= 3).


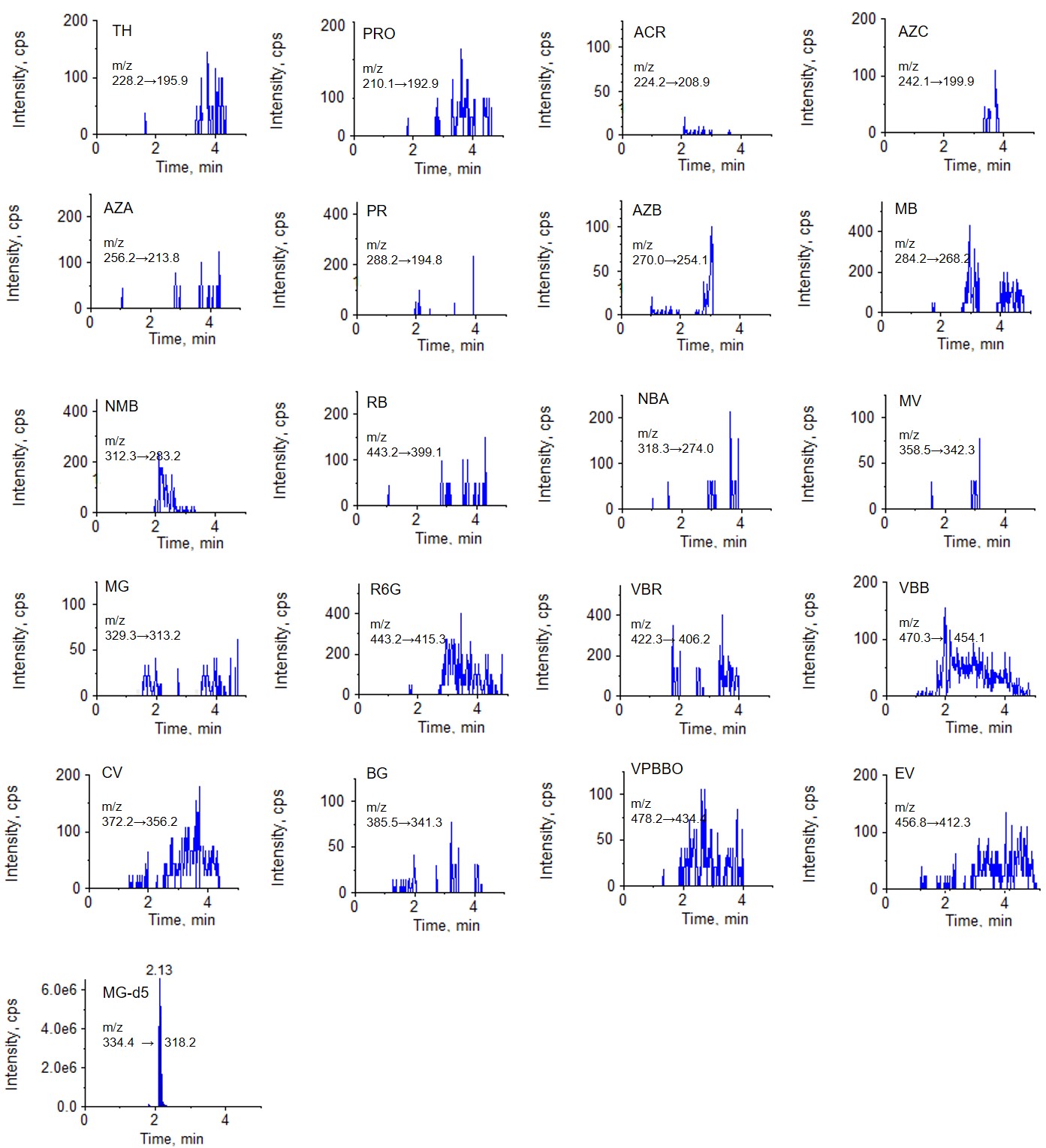


**Figure S5.** Multiple reaction monitoring (MRM) chromatograms of 20 pharmacologically active dyes: TH, PRO, ACR, AZC, AZA, PR, AZB, MB, NMB, RB, NBA, MV, MG, R6G, VBR, VBB, CV, BG, VPBBO, EV and an internal standard (MG-d5) in blank water sample.

**Table S1.** Selected parameters of heater gas (GS2), ion spray voltage (IS), interface temperature (TEM) used in additional tests (SET1-9) during ion source optimisation.

|  | **SET1** | **SET2** | **SET3** | **SET4** | **SET5** | **SET6** | **SET7** | **SET8** | **SET9** |
| --- | --- | --- | --- | --- | --- | --- | --- | --- | --- |
| **GS2 (PSI)** | 60 | 60 | 60 | 60 | 60 | 60 | 60 | 80 | 80 |
| **IS (V)** | 1000 | 1000 | 1000 | 1000 | 1500 | 2000 | 1500 | 1500 | 1500 |
| **TEM (°C)** | 400 | 500 | 600 | 700 | 600 | 600 | 500 | 500 | 600 |

selected ion source parameters: CUR=20, CAD – medium and GS1=40.

**Table S2.** Selected values for ion source parameters used for tandem mass spectrometry determination of 20 pharmacologically active dyes.

| **Ion source parameter** | **Selected value** |
| --- | --- |
| **curtain gas (CUR)** | 20 |
| **collision gas (CAD)** | MEDIUM |
| **nebulizer gas (GS1)** | 40 |
| **heater gas (GS2)** | 80 |
| **ion spray voltage (IS)** | 1500 |
| **temperature (TEM)** | 600 |

**Table S3.** Selected values for chromatographic separation of 20 pharmacologically active dyes.

| Chromatographic parameter | Selected value |
| --- | --- |
| **chromatography column:** | pentafluorophenyl (F5) analytical column, 1.7 µm, 2.1 × 100 mm |
| **temperature**  **of column** | 40°C |
| **mobile phase**  **(A):** | 0.05 M ammonium acetate buffer  (pH=3.5) |
| **mobile phase**  **(B):** | acetonitrile |
| **gradient elution settings:** | 0.0-0.2 min 10%, from 0.3-3.5 min 90%, 3.6-5.0 min 10% of the mobile phase B. |
| **mobile phase**  **flow:** | 0.4 ml/min |
| **injection**  **volume:** | 1. μl |

**Table S4.** Regression equations and coefficients of determination (R^2^) used in the method for determination of 20 pharmacological active dyes in water.

| **Analyte** | **Regression equation** | **R^2^** |
| --- | --- | --- |
| **malachite green (MG)** | y=33.7x+0.0275 | 0.9999 |
| **crystal violet (CV)** | y=94.6x+0.0145 | 0.9999 |
| **brilliant green (BG)** | y=92.2x+0.111 | 0.9992 |
| **ethyl violet (EV)** | y=84.6x+1.02 | 0.9996 |
| **methyl violet 2B (MV)** | y=19.9x+0.0603 | 0.9957 |
| **pararosaniline (PR)** | y=26.8x+0.0135 | 0.9998 |
| **victoria blue B (VBB)** | y=14.2x+0.135 | 0.9993 |
| **victoria blue R (VBR)** | y=21.5x+0.228 | 0.9981 |
| **victoria pure blue BO (VPBBO)** | y=54.3x+0.494 | 0.9988 |
| **methylene blue (MB)** | y=27.4x+0.0813 | 0.9989 |
| **azure A (AZA)** | y=21.3x+0.16 | 0.9991 |
| **azure B (AZB)** | y=30.2x+0.113 | 0.9993 |
| **azure C (AZC)** | y=20.5x+0.164 | 0.9991 |
| **thionine (TH)** | y=2.97x+0.0113 | 0.9993 |
| **new methylene blue (NMB)** | y=15.6x+0.0047 | 0.9994 |
| **nile blue A (NBA)** | y=35.5x+0.226 | 0.9988 |
| **acriflavine (ACR)** | y=15.5x+0.116 | 0.9997 |
| **proflavine (PRO)** | y=10.4x+0.119 | 0.9987 |
| **rhodamine B (RB)** | y=83x+0.0899 | 0.9985 |
| **rhodamine 6G (R6G)** | y=78.4x+0.0727 | 0.9990 |
